# Orthogonal Time Frequency Space Modulation Based on the Discrete Zak Transform

**DOI:** 10.3390/e24121704

**Published:** 2022-11-22

**Authors:** Franz Lampel, Hamdi Joudeh, Alex Alvarado, Frans M. J. Willems

**Affiliations:** Information and Communication Theory Lab, Signal Processing Systems Group, Department of Electrical Engineering, Eindhoven University of Technology, 5600 MB Eindhoven, The Netherlands

**Keywords:** orthogonal time frequency space modulation, discrete Zak transform, delay-Doppler channel, time-frequency dispersive channel, 6G

## Abstract

In orthogonal time frequency space (OTFS) modulation, information-carrying symbols reside in the delay-Doppler (DD) domain. By operating in the DD domain, an appealing property for communication arises: time-frequency (TF) dispersive channels encountered in high-mobility environments become time-invariant. OTFS outperforms orthogonal frequency division multiplexing (OFDM) in high-mobility scenarios, making it an ideal waveform candidate for 6G. Generally, OTFS is considered a pre- and postprocessing step for OFDM. However, the so-called Zak transform provides the fundamental relation between the DD and time domain. In this work, we propose an OTFS system based on the discrete Zak transform (DZT). To this end, we discuss the DZT and establish the input–output relation for time-frequency (TF) dispersive channels solely by the properties of the DZT. The presented formulation simplifies the derivation and analysis of the input–output relation of the TF dispersive channel in the DD domain. Based on the presented formulation, we show that operating in the DD incurs no loss in capacity.

## 1. Introduction

Motivated by challenges encountered in wireless communication over time-variant channels, such as Doppler dispersion or equalization, a new modulation technique termed orthogonal time frequency space (OTFS) was introduced in [1]. The driving idea behind OTFS is to utilize the delay-Doppler (DD) domain to represent information-carrying symbols. The interaction of the corresponding OTFS waveform with a time-frequency (TF) dispersive channel results in a two-dimensional convolution of the symbols in the DD domain ([2], [Section III-A]). OTFS utilizes the time-invariant channel interaction in the DD domain and outperforms orthogonal frequency division multiplexing (OFDM) in high-mobility scenarios, as shown in [1,2,3,4,5,6], making it an ideal waveform candidate for 6G.

Most of the literature on OTFS considers OTFS as a pre- and postprocessing technique for OFDM systems, as described in [3,5,7]. However, the *continuous* Zak transform provides a more fundamental relationship between the DD and time domain, as pointed out in [2] and studied in [8]. In principle, OTFS describes a time domain signal by its DD representations in a similar way to OFDM, which defines a signal in the TF domain. The difference between the DD and TF domains is that the TF domain allows a continuous-time signal to be described by a discrete number of coefficients in the TF domain [9]. On the other hand, the *continuous* Zak transform maps a continuous-time signal to continuous values in the Zak domain. In [8], a discretization of the Zak representation was achieved using time and bandwidth limitations on the signal, represented by a point in the DD domain. However, depending on the domain of the signal under study, different variants of the Zak transform exists. The discrete-time version is referred to as the discrete-time Zak transform (DTZT) and the discrete (and finite) version is the discrete Zak transform (DZT) [10]. The DTZT is discrete in the delay and continuous in the frequency domain, while the DZT is discrete in both the delay and Doppler domains. Thus, an alternative description of OTFS can be provided by the DZT, as we show in this work.

Another motivation for using the DZT can be found by considering OFDM. The fundamental concept of OFDM, that is, mapping symbols onto a set of orthogonal signals in the frequency domain, dates back to 1966 [11]. The success of OFDM is based on its efficient *digital* implementation to compute the discrete Fourier transform (DFT) [12]. Equivalently, OTFS can be efficiently implemented using the *discrete* Zak transform (DZT). The DZT itself is based on the DFT, which allows for efficient implementation as well. Implementations of OTFS which resemble the DZT have been studied previously, in [13], for example. However, the proposed systems is based on OFDM that adds a cyclic prefix (CP) to every OFDM symbol. The CP adds additional signaling overhead and results in a different channel interaction in the DD domain.

DZT-based OTFS is closely related to radar processing in a pulse Doppler radar. A pulse radar transmits a pulse train with uniformly spaced and identical pulses. Target motion introduces a phase shift for each pulse, which is utilized at the receiver to extract the velocity information of a radar target. To this end, the sampled signal is arranged in a two-dimensional grid, and a DFT is applied along the so-called slow time to extract the velocity information of a target; see ([14], [Chapter 17]) or ([15], [Chapter 3]) for details. This variant of Doppler processing is equivalent to the DZT. Similarly, the radar transmitter of such a pulse Doppler radar can be described by the inverse DZT, as demonstrated in [16]. The close connection to radar makes OTFS an ideal waveform for joint communication and sensing, which has been explored by [6], among others.

A fundamental treatment of OTFS based on the DZT is currently absent from the literature. The aim of this work is to close this gap in the literature by providing a complete treatment of OTFS based solely on the DZT. Therefore, we discuss the DZT and its properties, then we derive the input–output relationship for TF dispersive channels in the DD using the DZT and its properties. Our DZT-based approach provides an intuitive understanding of OTFS and drastically simplifies its analysis. Based on our analysis, we further show that the capacity in the DD domain is equivalent to the capacity of the time-variant channel in the time domain (Parts of this work were presented at the 2022 IEEE International Conference on Communications Workshops (ICC Workshops) [17]).

The remainder of the paper is organized as follows. In Section 2, we provide an introduction to the DZT covering all properties needed for OTFS. The signal model based on the DZT is described in Section 3. Based on the presented signal model, we further establish the input–output relationship of OTFS based on the DZT in Section 4. In Section 5, we establish the connection between the DD and the TF domain, which allows the implementation of OTFS by an OFDM system. In Section 6, we demonstrate that operating in the DD incurs no loss in capacity. Finally, our conclusions are presented in Section 7.

## 2. Discrete Zak Transform

The *continuous* Zak transform is a mapping of a continuous-time signal onto a two-dimensional function. Implicit usage of the Zak transform can be traced back to Gauss [18]; however, it was Zak who formally introduced the transform in [19], and after whom it was named. An excellent paper from a signal theoretical point of view was provided by Janssen [20]. Later on, Bölcskei and Hlawatsch [10] provided an overview of the discrete versions of the transform, namely, the discrete-time Zak transform and the *discrete* Zak transform. This section is devoted to the DZT and its properties, which we use to describe OTFS and to establish the input–output relation of the TF dispersive channel discussed in Section 3.

### 2.1. Definition and Relations

In the following discussion, we treat finite-length sequences of length *N* as one period of a periodic sequence with period *N*, which we express as a product N=KL with K,L∈N. Following the notation in [10], we use $Z_{x}^{\left(L,K\right)}\in C^{Z\times Z}$ to denote the DZT of a sequence $x\in C^{Z}$ with a period KL. The DZT of *x* is defined as follows ([10], Equation (30)):

(1) $$Z_{x}^{\left(L,K\right)}\left[n,k\right]≜\frac{1}{\sqrt{K}}\sum_{l=0}^{K-1}\underset{x^{\left(n,L\right)}\left[l\right]}{\underset{︸}{x[n+lL]}}e^{-j2\pi \frac{k}{K}l},\;n,k\in Z.$$

It follows from (1) that the DZT for a given *n* is the unitary discrete Fourier transform (DFT) of a subsampled sequence $x^{\left(n,L\right)}≜\{x^{\left(n,L\right)}[l]=x[n+lL]:l\in Z\}$. The variable *n* determines the starting phase of the downsampled sequence, whereas the variable *k* is the discrete frequency of its DFT. Thus, the variables *n* and *k* represent the time and frequency, respectively.

The *periodic* sequence *x* can be recovered from its DZT through the following sum relation:

(2) $$x\left[n\right]=\frac{1}{\sqrt{K}}\sum_{k=0}^{K-1}Z_{x}^{\left(L,K\right)}\left[n,k\right],$$

which follows from the definition of the DZT in (1) and the relation

(3) $$\sum_{k=0}^{K-1}e^{-j2\pi \frac{l}{K}k}=K\sum_{m=-\infty }^{\infty }\delta \left[l-mK\right],$$

where δ[n] denotes the Kronecker delta. We refer to (2) as the inverse discrete Zak transform (IDZT).

**Remark** **1**. *Depending on the period N of the sequence under consideration, different choices of K and L are possible. We indicate the particular choice of L and K in the superscript of the DZT notation we use ($Z_{x}^{\left(L,K\right)}$). If the choice is not important for the context, we drop the superscript for brevity of notation $\left(Z_{x}\right)$. Furthermore, the DZT is in general a complex-valued function. To illustrate the DZT, we often write the DZT in polar form, i.e.,*

(4) $$Z_{x}\left[n,k\right]=\left|Z_{x}[n,k]\right|e^{j\varphi_{x}\left[n,k\right]},$$

*where $\left|Z_{x}[n,k]\right|$ and $\varphi_{x}\left[n,k\right]$ represent the magnitude and the phase of $Z_{x}^{\left(L,K\right)}\left[n,k\right]$, respectively. We restrict the phase to the principal values, i.e., to the interval [−π,π).*

**Example** **1** (DZT). *Consider the N-periodic sequence g with elements*

(5) $$g\left[n\right]=\left\{\begin{array}{cc} f[n], & 0\leq n\leq L-1,\\ 0, & L\leq n\leq KL-1. \end{array}\right.$$

*The sequence is zero, except possibly for the first L samples, where it takes the value of an arbitrary sequence f. The second condition in (5) implies that only one nonzero addend (for l=0) exists in the summation (1). Thus, the elements of $Z_{g}$ for 0≤n≤L−1 and 0≤k≤K−1 are*

(6) $$Z_{g}\left[n,k\right]=\frac{1}{\sqrt{K}}f\left[n\right].$$

**Figure 1:**
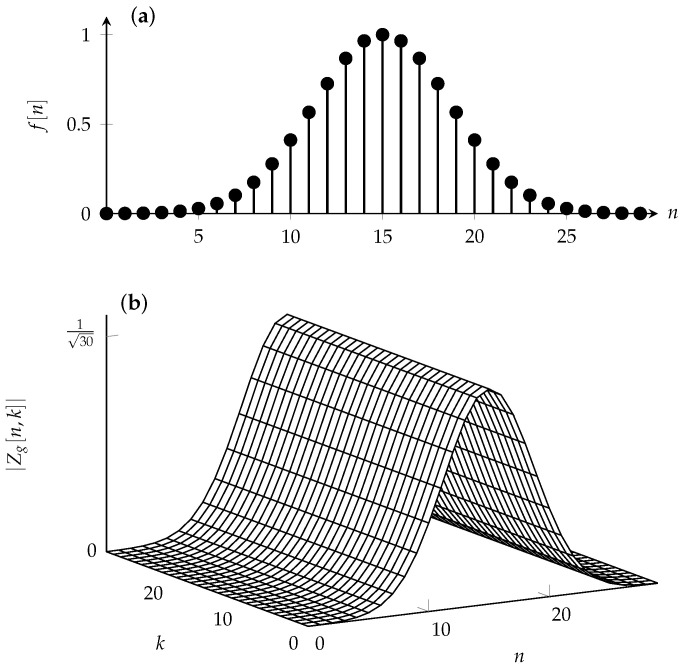
(**a**) Sequence $f\left[n\right]=e^{-\frac{1}{2}{\left(\frac{n-L/2}{\sigma L/2}\right)}^{2}}$ for σ=1/4, 0≤n≤L−1 and L=30. The sequence *g* has a period KL=900. (**b**) Magnitude of the discrete Zak transform (DZT) $Z_{g}$ with parameters K=30, L=30 in (6), for 0≤n≤L−1 and 0≤k≤K−1. The phase $\varphi_{g}\left[n,k\right]$ (not plotted) is zero for the presented values of *n* and *k*; see (6).

*Example for a sequence f and the corresponding magnitude of the DZT $Z_{g}$ are illustrated in Figure 1a,b, respectively.*

We express the period of the sequence *x* as a product KL with K,L∈N. This factorization ensures that the sequence can be decomposed into *L* subsampled sequences with period *K*. In general, the product KL is not uniquely defined, as different choices of *K* and *L* result in the same product. Independent of the period, two choices are always possible and provide interesting insights. First, the choice K=1 in (1) leads to

(7) $$Z_{x}^{\left(L,1\right)}\left[n,k\right]=x\left[n\right],$$

i.e., the elements of DZT for a specific *n* and any *k* are the elements of the sequence *x*. Second, the case L=1 results in

(8) $$Z_{x}^{\left(1,K\right)}\left[n,k\right]=\frac{1}{\sqrt{K}}\sum_{l=0}^{K-1}x\left[n+l\right]e^{-j2\pi \frac{k}{K}l}.$$

For n=0, we obtain

(9) $$Z_{x}^{\left(1,K\right)}\left[0,k\right]=X\left[k\right]$$

where $X\in C^{Z}$ is the unitary DFT of the sequence *x*, i.e.,

(10) $$X\left[k\right]≜\frac{1}{\sqrt{K}}\sum_{l=0}^{K-1}x\left[l\right]e^{-j2\pi \frac{k}{K}l}.$$

It follows from (8) that $Z_{x}^{\left(1,K\right)}\left[n,k\right]$ represents the DFT of the circular shifted sequence *x* with shift parameter *n*. Using the circular shift property of the DFT provided in ([21], Equation (3.168))

(11) $$x\left[n-n_{0}\right]\Leftrightarrow e^{-j2\pi \frac{k}{K}n_{0}}X\left[k\right],$$

we can express (8) equivalently as

(12) $$Z_{x}^{\left(1,K\right)}\left[n,k\right]=e^{j2\pi \frac{k}{K}n}X\left[k\right]=e^{j2\pi \frac{k}{K}n}Z_{x}^{\left(1,K\right)}\left[0,k\right].$$

Following the same approach used to obtain the DFT (9), we can obtain the inverse DFT (IDFT). Therefore, we consider (2) for the case L=1, which is

(13) $$x\left[n\right]≜\frac{1}{\sqrt{K}}\sum_{k=0}^{K-1}X\left[k\right]e^{j2\pi \frac{k}{K}n},$$

where (13) is obtained by substituting (12) in (2).

While the DZT $Z_{x}$ of a sequence *x* can be obtained from a sequence *x*, it can additionally be obtained from its DFT *X* in (9) through

(14) $$Z_{x}^{\left(L,K\right)}\left[n,k\right]=\frac{1}{\sqrt{L}}\sum_{l=0}^{L-1}X\left[k+lK\right]e^{j2\pi \frac{k+lK}{KL}n}.$$

**Proof.**  See Appendix A. □

Equivalently, using (1), we recognize (14) as

(15) $$Z_{x}^{\left(L,K\right)}\left[n,k\right]=e^{j2\pi \frac{n}{KL}k}Z_{X}^{\left(K,L\right)}\left[k,-n\right],$$

where $Z_{X}^{\left(K,L\right)}$ is the DZT of the DFT sequence *X*.

The corresponding inverse relation is

(16) $$X\left[k\right]=\frac{1}{\sqrt{L}}\sum_{n=0}^{L-1}Z_{x}^{\left(L,K\right)}\left[n,k\right]e^{-2\pi \frac{k}{KL}n}.$$

**Proof.**  See Appendix B. □

**Figure 2:**
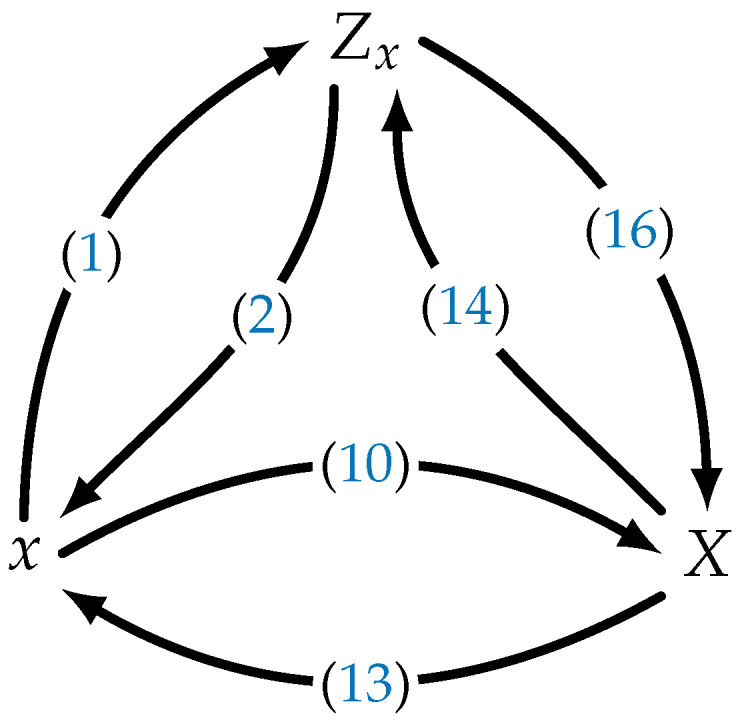
Different signal representations of a sequence *x* and its corresponding DZT $Z_{x}$ and DFT *X* transforms.

Figure 2 summarizes the relations between the sequence *x*, the DZT *Z_x_*, and the DFT *X*. Note that the DFT *X* can be obtained in two ways: either directly via (10) or indirectly using (1) and (16). The later approach resembles the Cooley–Tukey algorithm, which is a fast Fourier transform algorithm [10].

### 2.2. Properties of the DZT

The DFT *X* of a sequence *x* with length *K* is periodic with period *K*, i.e., X[k]=X[k+mK] with m∈Z; see (10). The DZT possess similar properties, as the DZT is the DFT of the downsampled sequence $x^{\left(n,L\right)}$; see (1). Consequently, the DZT is periodic in the frequency variable *k*, i.e.,

(17) $$Z_{x}^{\left(L,K\right)}\left[n,k+mK\right]=Z_{x}^{\left(L,K\right)}\left[n,k\right],\;m\in Z.$$

Using the circular shift property of the DFT in (11), we then have

(18) $$Z_{x}^{\left(L,K\right)}\left[n+mL,k\right]=e^{j2\pi \frac{k}{K}m}Z_{x}^{\left(L,K\right)}\left[n,k\right],\;m\in Z,$$

i.e., the DZT is periodic in *n* with a period *L* up to a complex factor $e^{j2\pi (k/K)m}$. The DZT is therefore said to be *quasi*-periodic with *quasi*-period *L*. Due to the periodicity properties in (17) and (18), the DZT is fully determined by the DZT for 0≤n≤L−1 and 0≤k≤K−1, which is referred to as the fundamental rectangle [10].

The *quasi*-periodicity in (18) can be utilized to express the IDZT in (2) as follows:

(19) $$x\left[n+lL\right]=\frac{1}{\sqrt{K}}\sum_{k=0}^{K-1}Z_{x}^{\left(L,K\right)}\left[n,k\right]e^{j2\pi \frac{k}{K}l}.$$

Here, we express the index of the sequence as sum of the form n+lL with 0≤n≤L−1 and l∈Z. Because the fundamental rectangle fully determines the DZT $Z_{x}$, we restrict ourselves to this fundamental rectangle when plotting the DZT. In fact, this is what is done in Figure 1b.

**Example** **2** (IDZT). *Consider the DZT defined by a single nonzero coefficient on the fundamental rectangle of size 4×6 and provided by*

(20) $$Z_{x}^{\left(4,6\right)}\left[n,k\right]=\delta \left[n\right]\delta \left[k\right].$$

**Figure 3:**
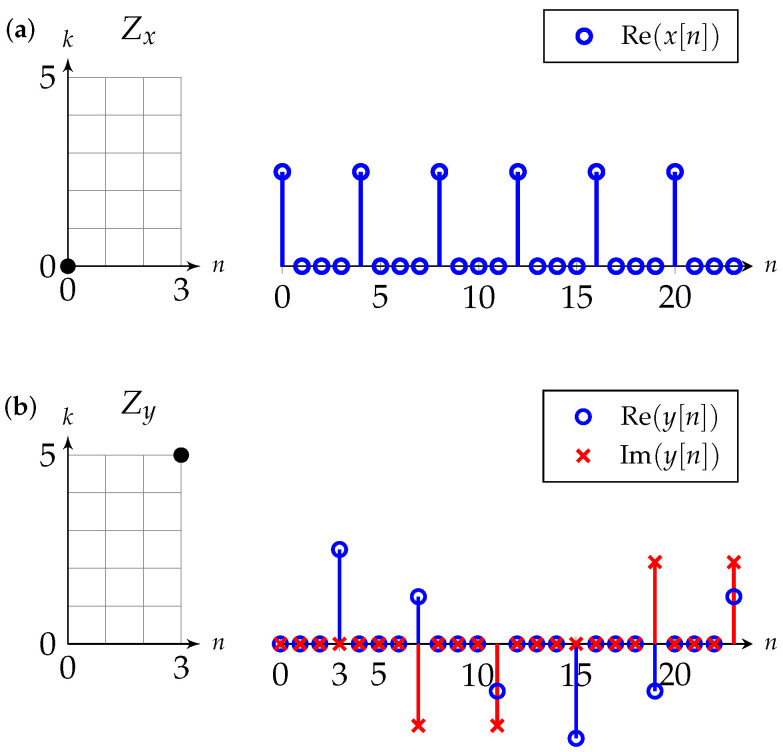
Two examples of DZTs (left) defined by a single nonzero coefficient on the fundamental rectangle (indicated by a dot) and the corresponding sequences (right) for (**a**) the DZT in (20) and (**b**) the DZT in (22).

*The fundamental rectangle and the DZT in (20) are illustrated in Figure 3a (left). One period of the sequence x obtained through (19) is*

(21) $$x\left[n\right]=\frac{1}{\sqrt{6}}\sum_{l=0}^{K-1}\delta \left[n-6l\right],$$

*i.e., a train of real Kronecker deltas starting at n=0 with spacing L=6, as shown in Figure 3a (right). Now, consider the DZT*

(22) $$Z_{y}^{\left(4,6\right)}\left[n,k\right]=\delta \left[n-3\right]\delta \left[k-5\right],$$

*which is shown in Figure 3b. One period of the corresponding sequence y is*

(23) $$y\left[n\right]=\frac{1}{\sqrt{6}}\sum_{l=0}^{K-1}\delta \left[n-3-6l\right]e^{j2\pi \frac{5}{6}l}$$

*and is shown in Figure 3b. When compared to x, the sequence y is delayed by three samples and modulated with a discrete frequency k=5.*

*In fact, a single coefficient at $Z_{x}\left[n,k\right]$ maps onto a sequence*

(24) $$v_{n,k}\left[n^{\prime }\right]=\frac{1}{\sqrt{K}}\sum_{l=0}^{K-1}\delta \left[n^{\prime }-n+lL\right]e^{j2\pi \frac{k}{K}l}.$$

*The set of sequence $\left\{v_{n,k}:0\leq n\leq L-1,0\leq k\leq K-1\right\}$ forms an orthonormal basis and $Z_{x}\left[n,k\right]$ are the* expansion coefficients *of a sequence x with respect to this orthonormal basis. We use this fact in Section 3, where we define a sequence by its corresponding DZT in the same way as OFDM defines the symbols in the DFT domain.*

Using the *quasi*-periodicity, we can further find that the elementwise product of a DZT $Z_{x}$ with the complex conjugate DZT $Z_{y}^{*}$ is periodic in *n* and *k*. Motivated by this periodicity, we apply a two-dimensional DFT, which turns out to be [10,22]

(25) $$\sum_{n=0}^{L-1}\sum_{k=0}^{K-1}Z_{x}\left[n,k\right]Z_{y}^{*}\left[n,k\right]e^{j2\pi \left(\frac{m}{K}k-\frac{l}{L}n\right)}=\langle x,y_{m,l}\rangle ,$$

where $y_{m,l}≜y\left[n-mL\right]e^{j2\pi (l/L)n}$. Here, 〈·,·〉 is the inner product, defined as

(26) $$\langle x,y\rangle =\sum_{n=0}^{N-1}x\left[n\right]y^{*}\left[n\right].$$

Note that the Fourier kernel $e^{j2\pi \left(\frac{m}{K}k-\frac{l}{L}n\right)}$ in (25) has opposed signs for the two individual dimensions. Therefore, the two-dimensional discrete Fourier transform in (25) is usually referred to as the inverse *symplectic* finite Fourier transform (ISFFT).

**Proof.**  See Appendix C. □

The inverse relation is provided by

(27) $$Z_{x}\left[n,k\right]Z_{y}^{*}\left[n,k\right]=\frac{1}{KL}\sum_{m=0}^{K-1}\sum_{l=0}^{L-1}\langle x,y_{m,l}\rangle e^{-j2\pi \left(\frac{k}{K}m-\frac{n}{L}l\right)},$$

which follows from applying the corresponding two-dimensional inverse transform on both sides of (25). The transform of the right-hand side of (27) is referred to as the symplectic finite Fourier transform (SFFT). The relations (25) and (27) provide a useful tool when considering the OTFS overlay for OFDM in Section 5.

### 2.3. Signal Transform Properties

Here, we list three signal transform properties that we use later when studying OTFS. A comprehensive overview of signal transform properties can be found in ([10], Table VII). Let *x*, *y*, and *z* be sequences with the same periods and let $Z_{x}$, $Z_{y}$, and $Z_{z}$ be their respective DZTs. Then, the following properties hold:

1. *Shift:* Let *y* be the shifted version of *x*, i.e., y[n]=x[n−m]; then,
  (28) $$Z_{y}\left[n,k\right]=Z_{x}\left[n-m,k\right].$$
  A shift in the sequence causes a shift in the corresponding DZT. The proof follows from the definition of the DZT (1). For shifts of multiples of *L*, i.e., m=lL with l∈Z, we further have
  (29) $$Z_{y}\left[n,k\right]=e^{-j2\pi \frac{k}{K}m}Z_{x}\left[n,k\right],$$
  which follows from the *quasi*-periodicity of the DZT in (18).
2. *Modulation:* Let z=x·y be the elementwise product of *x* and *y*, i.e., z[n]=x[n]y[n]. Then,
  (30) $$Z_{z}\left[n,k\right]=\frac{1}{\sqrt{K}}\sum_{l=0}^{K-1}Z_{x}\left[n,l\right]Z_{y}\left[n,k-l\right],$$
  i.e., the DZT of the element-wise multiplication is a scaled convolution with respect to the variable *k*.

**Proof.**  See Appendix D. □

3. *Circular Convolution:* Consider z=x⊛y, i.e., the circular convolution of *x* and *y*. Then, the DZT $Z_{z}$ is
  (31) $$Z_{z}\left[n,k\right]=\sqrt{K}\sum_{m=0}^{L-1}Z_{x}\left[m,k\right]Z_{y}\left[n-m,k\right],$$
  i.e., the DZT of a circular convolution is the scaled convolution with respect to the variable *n* up to a constant.

**Proof.**  See Appendix E. □

The shift property in (28) together with the *quasi*-periodicity in (18) has another important implication. In OTFS, as we show in Section 3, the received signal includes a superposition of delayed sequences that, in general, are not multiples of *L*. We discuss this further in Example 3.

**Example** **3** (Shifted DZT). *Consider a DZT $Z_{h}$ with elements*

(32) $$Z_{h}\left[n,k\right]=Z_{g}\left[n-10,k\right],$$

*which is a shifted version of the DZT $Z_{g}$ in Figure 1b of Example 1. To evaluate the DZT $Z_{h}$ within the fundamental rectangle, we first make the observation that any index n can be expressed as n=i+mL with m=⌊n/L⌋, where ⌊n/L⌋ denotes the greatest integer less than or equal to n/L. In this example, the indices n=0 to 9 of $Z_{h}$ correspond to the indices n=−10 to −1 of $Z_{g}$. Expressing the latter indices in terms of i and m, we know m=−1 and i from 20 to 29. Thus, by the* quasi*-periodicity property in (18), we have that $Z_{h}\left[n,k\right]=e^{-j2\pi k/K}Z_{g}\left[n+20,k\right]$ for 0≤n≤9. On the other hand, the indices of 10≤n≤29 of $Z_{h}\left[n,k\right]$ correspond to the indices 0≤n≤19 of $Z_{g}\left[n,k\right]$. Therefore, m=0 and $Z_{h}$ is the shifted DZT $Z_{g}$ within the fundamental rectangle. Thus,*

(33) $$Z_{h}\left[n,k\right]=\left\{\begin{array}{cc} e^{-j2\pi \frac{k}{K}}Z_{g}\left[n+20,k\right], & 0\leq n\leq 9,\\ Z_{g}\left[n-10,k\right], & 10\leq n\leq 29, \end{array}\right.$$

**Figure 4:**
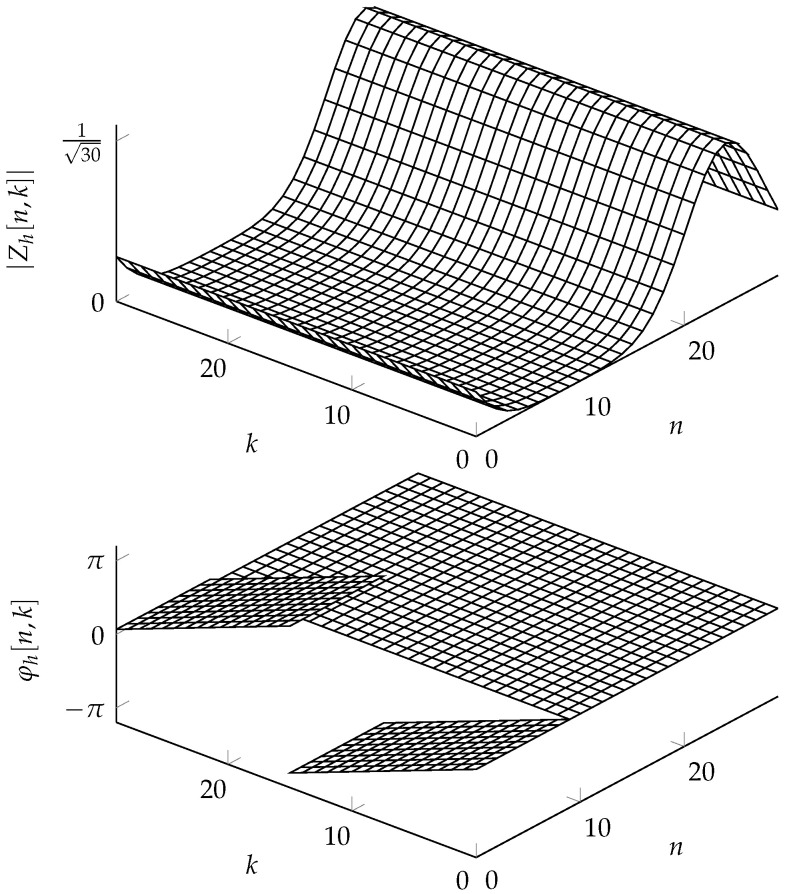
The DZT $Z_{h}\left[n,k\right]=Z_{g}\left[n-10,k\right]$ in Example 3, with $Z_{g}\left[n,k\right]$ being the DZT of Figure 1. The shift of the DZT with respect to *n* causes a circular shift of the magnitude $|Z_{g}\left[n,k\right]|$ of the DZT (**top**). The phase $\varphi_{h}\left[n,k\right]$ experiences an additional linear phase for indices smaller than 10 (**bottom**).

*or more generally, $Z_{h}\left[n,k\right]=e^{j2\pi (k/K)\lfloor (n-10)/L\rfloor }Z_{g}\left[{\left(n-10\right)}_{L},k\right]$. The DZT $Z_{h}$ is depicted in Figure 4, which illustrates different phase behaviors as well.*

## 3. System Model

**Figure 5:**
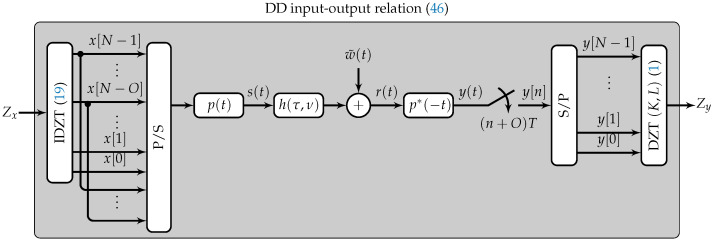
OTFS system model considered in this work. The IDZT maps a sequence consisting of the symbols defined in the DD domain to a discrete sequence. A CP is added by copying the last *O* samples. The resulting sequence *x* is converted to a serial stream by a parallel-to-serial converter (P/S) before being mapped onto a pulse p(t) and sent over a noisy TF-dispersive channel h(τ,ν). At the receiver, a sampled matched filter is applied before the serial stream is converted to a parallel stream by a serial-to-parallel (S/P) converter. Lastly, the sequence *y* is mapped to the DD domain using the DZT. The DD input–output relationship is provided by (46) and Theorem 1.

In this section, we use the IDZT/DZT to map the symbols in the DD domain directly to a time domain sequence and vice versa. We consider a pulse-amplitude modulation (PAM) system to map the discrete symbols onto continuous pulses, as schematically shown in Figure 5. This approach allows for the digital implementation of OTFS similar to the PAM implementation of OFDM presented in ([23] Chapter 6.4.2).

### 3.1. Transmitter

Similar to OFDM, which defines symbols in the frequency domain, OTFS defines K×L symbols on the fundamental rectangle in the Zak domain. The symbols in the Zak domain are mapped to a sequence in the time domain using the IDZT in (19). Prior to modulation, a CP of length *O* is added by copying the last *O* samples and inserting them at the beginning of the sequence (see Figure 5). As we show later, the CP turns the linear convolution of the channel into a circular convolution, allowing us to use the circular convolution property (47) of the DZT. The elements of the sequence *x* are then mapped onto time-shifted pulses p(t) using PAM. The transmitted signal is provided as follows:

(34) $$s\left(t\right)=\sum_{n=0}^{N+O-1}x\left[n-O\right]p\left(t-nT\right),$$

where *T* is the modulation interval and p(t) is a square-root Nyquist pulse. Note that (34) is equivalent (up to the CP) to (21) of [8]. However, by considering the DZT and PAM, no discretization of the continuous Zak transform is required. Moreover, considering the class of Nyquist pulses in the modulation allows for more freedom in controlling the interference in the delay domain.

**Remark** **2**. *In Section 2.1, we discussed the implications of the choice of the parameters K and L for the DZT. Similarly, the choice of K and L influences the OTFS system under study. For the case K=1, the symbols of $Z_{x}$ are arranged on a line along the delay axis. The IDZT does not alter the sequence and can be skipped; see (7). Thus, the system is a single carrier system. On the other hand, for L=1, the symbols $Z_{x}^{\left(L,K\right)}\left[n,k\right]$ are arranged along the Doppler axis. The IDZT is simply the IDFT (see (13)), and (34) becomes an OFDM signal as in ([23] Chapter 6.4.2).*

### 3.2. Channel Model

We now consider TF dispersive channels and model the received signal as follows ([24] Chapter 1.3.1):

(35) $$r\left(t\right)=\int_{-\infty }^{\infty }\int_{-\infty }^{\infty }h\left(\tau ,\nu \right)s\left(t-\tau \right)e^{j2\pi \nu t}d\tau d\nu +\tilde{w}\left(t\right)$$

where h(τ,ν) is the so-called DD spreading function. The complex noise $\tilde{w}(t)$ is assumed to be white and Gaussian with power spectral density $N_{0}$. We model the channel by *P* discrete scattering objects. Each scattering object is associated with a path delay $\tau_{p}$, a Doppler shift $\nu_{p}$, and a complex attenuation factor $\alpha_{p}$. Thus, the spreading function h(τ,ν) becomes

(36) $$h\left(\tau ,\nu \right)=\sum_{p=0}^{P-1}\alpha_{p}\delta \left(\tau -\tau_{p}\right)\delta \left(\nu -\nu_{p}\right).$$

Substituting (36) in (35) yields

(37) $$r\left(t\right)=\sum_{p=0}^{P-1}\alpha_{p}s\left(t-\tau_{p}\right)e^{j2\pi \nu_{p}t}+\tilde{w}\left(t\right),$$

i.e., the received signal is a superposition of scaled, delayed, and Doppler-shifted replicas of the transmitted signal. The Doppler shift is provided by $\nu_{p}=v_{p}f_{p}/c$, where $v_{p}$, *f_c_*, and *c* are the relative velocity of the *p*th scattering object, the carrier frequency, and the speed of light, respectively. The length of the CP in (34) is chosen such that *OT* is larger than or equal to the maximum delay.

**Remark** **3**. *In the channel model in (36), it is assumed that the individual delays are independent of the absolute time. Strictly speaking, this is not the case, as the movement of a reflector affects the delay. However, (36) holds as long as the signal length NT is chosen such that the delay does not change significantly.*

Substituting (34) in (37), the received signal is

(38) $$r\left(t\right)=\sum_{p=0}^{P-1}\alpha_{p}\sum_{n=0}^{N+O-1}x\left[n-O\right]p\left(t-nT-\tau_{p}\right)e^{j2\pi \nu_{p}t}+\tilde{w}\left(t\right).$$

### 3.3. Receiver

At the receiver, a matched filter with impulse response $p^{*}\left(-t\right)$ is applied. The output of the matched filter y(t) is

(39) $$y\left(t\right)=\sum_{p=0}^{P-1}\alpha_{p}\sum_{n=0}^{N+O-1}x\left[n-O\right]\int_{-\infty }^{\infty }p\left(\tau -nT-\tau_{p}\right)e^{j2\pi \nu_{p}\tau }p^{*}\left(\tau -t\right)d\tau +w\left(t\right),$$

where w(t) is the filtered noise. Assuming that the pulse bandwidth is much larger than the maximum Doppler shift, we can approximate the integral in (39) as $e^{j2\pi \nu_{p}\left(nT+\tau_{p}\right)}h\left(t-nT-\tau_{p}\right)$, where h(t) is the corresponding Nyquist pulse. The output of the matched filter is then

(40) $$y\left(t\right)\approx \sum_{p=0}^{P-1}\alpha_{p}\sum_{n=0}^{N+O-1}x\left[n-O\right]e^{j2\pi \nu_{p}\left(nT+\tau_{p}\right)}h\left(t-nT-\tau_{p}\right)+w\left(t\right).$$

The matched filter output is sampled every *OT* seconds and with an offset of *T* to discard the CP. The sampled signal y[m]=y((m+)T) is

(41) $$\begin{array}{c} y\left[n\right]=\sum_{p=0}^{P-1}\alpha_{p}\sum_{m=-O}^{N-1}x\left[m\right]e^{j2\pi \frac{k_{p}}{KL}m}h_{\tau_{p}}\left[n-m\right]+w\left[n\right], \end{array}$$

where $h_{\tau_{p}}\left[n\right]=h\left(nT-\tau_{p}\right)$ is the sampled Nyquist pulse and w[m] are independent and identically distributed (i.i.d.) complex zero-mean Gaussian random variables with variance $N_{0}$. To shorten the notation, we combine the constant phase terms $e^{j2\pi \nu_{p}\tau_{p}}$ with the channel gain $\alpha_{p}$ in (41). Furthermore, we express $\nu_{p}$ as a multiple of the Doppler resolution, which we define as

(42) Δν≜1/(KLT),

i.e., $\nu_{p}=\Delta \nu k_{p}$.

We can bound the interval for which h(t) is significantly different from zero (for sufficient large *L*) to ±LT/2. Thus, we can express $h_{\tau_{p}}[n]$ as

(43) $$h_{\tau_{p}}\left[n\right]=\left\{\begin{array}{cc} h(nT-\tau_{p}), & \mathrm{for}-\frac{LT}{2}\leq nT-\tau_{p}<\frac{LT}{2},\\ 0, & \mathrm{else}. \end{array}\right.$$

The CP allows the linear convolution in (41) to be approximated by a circular convolution; the sample y[n] is then provided by

(44) $$y\left[n\right]=\sum_{p=0}^{P-1}\alpha_{p}y_{p}\left[n\right]+w\left[n\right],$$

where

(45) $$y_{p}\left[n\right]=\sum_{m=0}^{KL-1}x\left[m\right]e^{j2\pi \frac{k_{p}}{N}m}h_{\tau_{p}}\left[n-m\right].$$

Here, $h_{\tau_{p}}$ is periodicized over a period KL, i.e., $h_{\tau_{p}}[n]=h_{\tau_{p}}[n+KL]$. In a last step, the receiver computes the DZT of the sequence y[m] before subsequent processing takes place.

## 4. Delay Doppler Input–Output Relationship

To express the input–output relationship in the DD domain for the system presented in Figure 5, we first note that the DZT is a linear transform; as such, we can write the DZT of (44) as

(46) $$Z_{y}\left[n,k\right]=\sum_{p=0}^{P-1}\alpha_{p}Z_{y_{p}}\left[n,k\right]+Z_{w}\left[n,k\right],$$

where is the DZT of sequence $y_{p}$ described in (45) and $Z_{w}\left[n,k\right]$ is the DZT of the noise. The elements of $Z_{w}\left[n,k\right]$ are i.i.d. zero-mean Gaussian random variables with variance $N_{0}$. This follows from the fact that the DZT is a unitary transform ([10], Section VI).

For the signal model of a single reflector in (45), we provide the following result for the input–output relationship in the DD domain for the OTFS system described in Section 3.

**Theorem** **1**. *Considering the fundamental rectangle $\in C^{L\times K}$ of complex symbols in the DD domain, the input–output relation for OTFS transmission over a time-frequency selective channel for a single reflector is*

(47) $$Z_{y_{p}}\left[n,k\right]=\sum_{m=0}^{L-1}\left(\sum_{l=0}^{K-1}Z_{x}\left[m,l\right]Z_{\nu_{p}}\left[m,k-l\right]\right)Z_{\tau_{p}}\left[n-m,k\right],$$

*where $Z_{\tau_{p}}$ and $Z_{\nu_{p}}$ are the delay and Doppler spreading functions, respectively. The delay spreading function $Z_{\tau_{p}}$ is the DZT of the shifted and sampled impulse $h_{\tau_{p}}\left[n\right]$ in (43), and the Doppler spreading functions is provided as follows:*

(48) $$Z_{\nu_{p}}\left[n,k\right]=\frac{1}{\sqrt{K}}e^{j2\pi \frac{k_{p}}{KL}n}e^{-j\pi \frac{K-1}{K}\left(k-k_{p}\right)}\frac{\sin\left(\pi (k-k_{p})\right)}{\sin\left(\frac{\pi }{K}\left(k-k_{p}\right)\right)}.$$

**Proof.**  See Appendix F. □

To illustrate the spreading of a single symbol in the DD domain, we consider the following example. Let L=K=30 and

(49) $$Z_{x}\left[n,k\right]=\left\{\begin{array}{cc} 1 & \mathrm{for}\;n=k=L/2,\\ 0 & \mathrm{else}. \end{array}\right.$$

**Figure 6:**
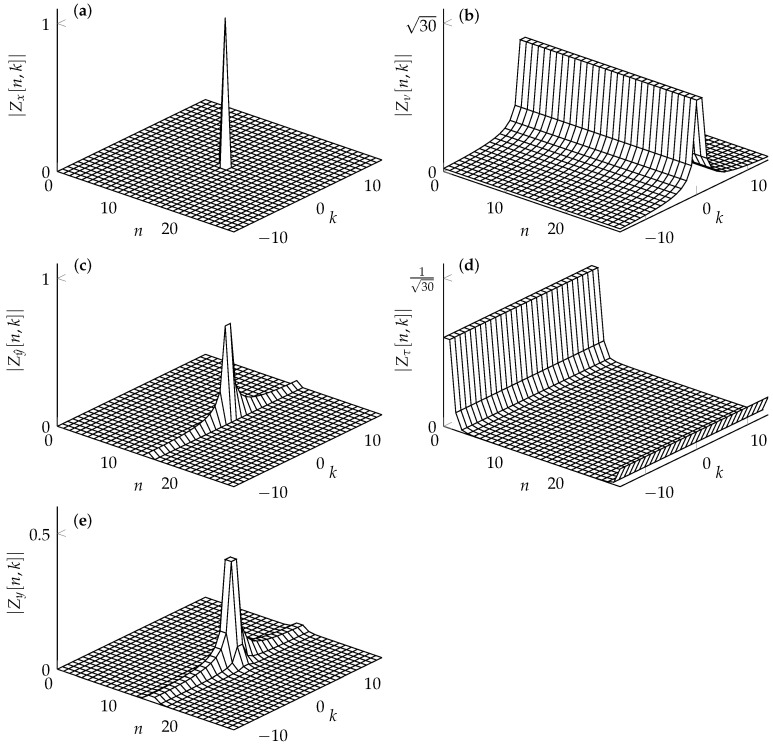
Example of the spread of a symbol (**a**) in the DD domain due to fractional delay and Doppler shift. The spread can be first evaluated in the Doppler domain (**c**) using the Doppler spreading function in (**b**). The spread symbol in the Doppler domain is further spread in the delay by the the delay spread function in (**d**). The overall spread in the DD domain is shown in (**e**).

The fundamental rectangle with the only nonzero element is presented in Figure 6a. Furthermore, assume that τ=0.5T and ν=0.5Δν. Note that this example causes the maximum spread of a single symbol in the DD domain. We can visualize the spreading of the symbol defined in (49) in two steps. Therefore, we define $Z_{\hat{y}}$ as the DZT resulting from the inner convolution in (47), presented in Figure 6b, with respect to the Doppler index *k*. The resulting spread of the nonzero symbol is visualized in Figure 6c. Finally, the symbol that has been spread in the Doppler domain is spread in the delay domain by the delay spreading function $Z_{\tau }$, which is illustrated in Figure 6d. Note that due to the limited support of $h_{\tau }$ (see (43)), the magnitude of $Z_{\tau }$ is independent of the index *k*. The resulting spread of the nonzero symbol in the DD domain is shown in Figure 6e.

For the particular case of $\tau_{p}=n_{p}T$ with $n_{p}=0,1,\ldots ,O-1$ and $\nu_{p}=k_{p}/\left(KLT\right)$ with $k_{p}\in Z$, $Z_{y_{p}}$ simplifies to

(50) $$Z_{y_{p}}\left[n,k\right]=e^{j2\pi \frac{k_{p}}{KL}\left(n-n_{p}\right)}Z_{x}\left[n-n_{p},k-k_{p}\right],$$

i.e., the received symbols $Z_{y_{p}}$ are in the DD domain displaced symbols $Z_{x}$.

**Figure 7:**
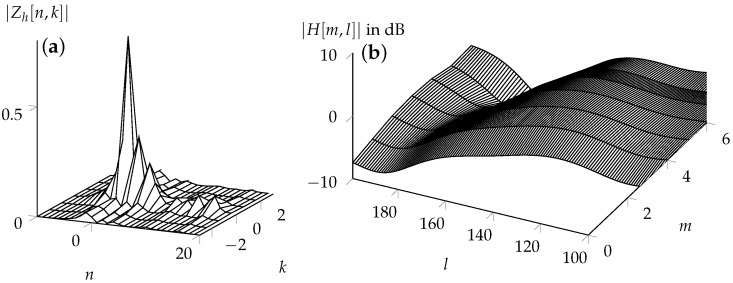
Two different representations of the time-variant channel: (**a**) DD representation and (**b**) TF representation. The DD domain representation is only nonzero for a small part of the domain, and stays constant throughout the transmission. On the other hand, the TF domain representation of the channel in the TF domain changes with respect to the time, and therefore needs to be tracked.

Theorem 1 shows that the channel interaction with the symbols in the DD domain is time-invariant, neglecting the additional phase terms due to the quasi-periodicity and modulation. The invariance is helpful in the detection of the symbols. Consider a TDL-C channel with a delay spread of 300 ns, a carrier frequency of 4 GHz, and a maximum velocity of 120 kmph. Furthermore, assume an OTFS system with K=7 and L=600 and 1/T=9 MHz. The channel response $Z_{h}\left[n,k\right]=\sum_{l=0}^{K-1}Z_{\nu_{p}}\left[n,k-l\right]Z_{\tau_{p}}\left[n,k\right]$ in the DD domain is illustrated in Figure 7a. The magnitude of this channel stays approximately constant throughout the entire transmission of an OTFS frame. Figure 7b illustrates the equivalent OFDM channel. The variation of the channel along the subcarrier index k as well along the time index n can be seen. To keep track of the channel, additional pilots need to be used, and these cannot be used for communication.

In addition to constant channel interaction, OTFS offers the advantage of a concise and sparse channel description compared to OFDM. In an OFDM system, the channel coefficient for each subcarrier must be estimated for subsequent symbol detection. In contrast, for symbol detection in an OTFS system, knowledge of the interference introduced by each reflector is sufficient. The sparsity can be seen in Figure 7; the support of $|Z_{h}\left[n,k\right]|$ is limited to a small area, while the channel transfer function changes with each subcarrier and time index, that is, *l* and *m*, respectively.

**Remark** **4**. *The discrete two-dimensional convolution in (46) can be equivalently expressed in the form*

(51) y=Hx+w,

*where y, x, and w are the vectorized DTZs $Z_{y}$, $Z_{x}$, and $Z_{w}$, respectively. The vectors are all of length KL. The matrix $H\in C^{KL\times KL}$ describes the intersymbol interference in the DD domain. Because $Z_{\tau_{p}}$ and $Z_{\nu_{p}}$ have small support in the DD domain, the corresponding matrix H is sparse. The matrix-vector formulation of the input–output relationship is the basis for many works on OTFS; for example, see [5,6].*

## 5. OTFS Overlay for OFDM

Currently, orthogonal frequency division multiplexing (OFDM) is the dominant modulation scheme in wireless communication. For example, it is used in 5G and in several 802.11 standards. This section shows that DFT-based ODFM can be used for OTFS modulation and demodulation. In this context, OTFS is considered a pre- and postprocessing step for the OFDM system.

To derive the pre- and postprocessing step, we first derive an alternative way to compute the DZT. For this purpose, we consider (27). If we choose the sequence *y* such that its DZT $Z_{y}\left[n,k\right]=1$, then we can obtain the DZT $Z_{x}$ through the right-hand side of (27). The *N* periodic sequence *y* with DZT $Z_{y}\left[n,k\right]=1$ is

(52) $$y\left[n\right]=\left\{\begin{array}{cc} \sqrt{K}, & 0\leq n\leq L-1,\\ 0, & \mathrm{elsewhere}. \end{array}\right.$$

With this particular choice of *y*, we recognize the inner product on the right-hand side of (27) as

(53) $$\langle x,y_{m,l}\rangle =\sqrt{K}\sum_{n=0}^{L-1}x\left[n+mL\right]e^{j2\pi \frac{l}{L}n},$$

which is the scaled *L*-point DFT of the samples x[n] for mL≤n≤(m+1)L−1. If we define

(54) $$a_{m,l}≜\langle x,y_{m,l}\rangle ,$$

for 0≤m≤K−1 and 0≤l≤L−1, then the DZT of *x* is obtained through

(55) $$Z_{x}\left[n,k\right]=\frac{1}{KL}\sum_{m=0}^{K-1}\sum_{l=0}^{L-1}a_{m,l}e^{-j2\pi \left(\frac{k}{K}m-\frac{n}{L}l\right)},$$

i.e., by the SFFT of the coefficients $a_{m,l}$. Note that the set $a_{m,l}$ represents the Gabor expansion coefficients for the choice of a rectangular analysis window (see [25], Section 4), and thus a mixed TF representation of the sequence *x*.

The coefficients $a_{m,l}$, on the other hand, are obtained from $Z_{x}^{\left(L,K\right)}\left[n,k\right]$ using (25):

(56) $$a_{m,l}=\sum_{n=0}^{L-1}\sum_{k=0}^{K-1}Z_{x}\left[n,k\right]\left[n,k\right]e^{j2\pi \left(\frac{k}{K}m-\frac{n}{L}l\right)}.$$

The samples of the sequence *x* for mL≤n≤(m+1)L−1 are obtained as follows:

(57) $$x\left[n+mL\right]=\frac{1}{\sqrt{K}L}\sum_{l=0}^{L-1}a_{m,l}e^{j2\pi \frac{l}{L}n},$$

which is the *L*-point IDFT of the coefficients *a_m,l_* for a fixed *m*. Thus, the DZT (IDZT) can be implemented by consecutive execution of the DFT (IDFT) and the SFFT (ISFFT).

The above-described two-step approach for the calculation of the DZT and IDZT can be used to implement OTFS using OFDM hardware, which is typically based on the IDFT/DFT (see ([26], Section 19.3), ([23], Section 6.4.2), ([27] Section 12.4.3), or ([28], Section 4.6)) by extending the transmitter and receiver by the ISFFT and SFFT, respectively. The coefficients *a_m,l_* then represent the coefficient in the TF domain. The index *m* refers to the *m*th OFDM symbol in the time domain, and *l* is the corresponding subcarrier index. Note that for the DZT, the parameter *L* the grid size in the delay domain. For DFT-SFFT implementation, on the other hand, *L* defines DFT size, which defines the number of points in the frequency domain. Thus, an L×K grid in the DD domain translates to a K×L grid in the TF domain.

**Remark** **5**. *In CP-OFDM, a CP is added for each OFDM symbol by copying the last *O* samples of an OFDM symbol and inserting them in front of the corresponding OFDM symbol with length L. This symbol-wise CP is not required in the OFDM implementation of OTFS. Instead, a single CP is added by copying the last *O* samples of the entire sequence and inserting them in front of the sequence.*

## 6. DD Channel Capacity

The input–output relationship in (41) is equivalently expressed as

(58) $$y\left[n\right]=\sum_{m\in L}h\left[n,m\right]x\left[n-m\right]+w\left[n\right],$$

where h[n,m] is the time-variant multi-tap channel response at time instance *n* and L is the support of h[n,m] in *m*. This channel response is deterministic and periodic (considering $k_{p}\in Q$) with some finite period *M*, i.e., h[n,m]=h[n+bM,m] for any n∈{1,2,…,M} and b∈Z. Upon using the channel *N* times, the input output relationship can be written in the following vector form:

(59) $$Y_{N}=H_{N}X_{N}+W_{N},$$

where $X_{N}$ is the input block, $Y_{N}$ is the corresponding output block, $W_{N}$ is the block of noise samples (all column vectors), and $H_{N}$ is the channel (convolution) matrix constructed from the time-varying channel response h[n,m].

The above channel can be shown to be *information-stable* (see Section 3.9 in [29]); hence, its capacity is provided by the following multi-letter limiting expression [30]:

(60) $$C=\lim_{N\to \infty }\underset{f_{X_{N}}}{\sup}\frac{1}{N}I\left(X_{N};Y_{N}\right),$$

where $f_{X_{N}}$ is the multi-letter input distribution for block length *N*. For each block length *N*, the corresponding mutual information term in (60) is maximized by a Gaussian input [31]; hence, the capacity is provided by

(61) $$C=\lim_{N\to \infty }\max_{Q_{N}:\mathrm{tr}\left(Q_{N}\right)\leq NP}\frac{1}{N}\log\det\left(\frac{1}{\sigma^{2}}H_{N}Q_{N}H_{N}^{H}+I_{N}\right).$$

Let $H_{N}=U_{N}\Sigma_{N}V_{N}^{H}$ be the SVD of $H_{N}$. Then, the optimal input covariance matrix is provided by $Q_{N}=V_{N}D_{N}V_{N}^{H}$, where $D_{N}$ is a diagonal matrix obtained using water-filling [31]. The capacity-achieving strategy is characterized by a sequence ${\left\{Q_{N}\right\}}_{N\in N}$.

In case we do not wish to use the channel response matrix in the construction of input sequences, we may add the restriction that the multi-letter input distribution must be isotropic. In this case, we simply have $Q_{N}=PI_{N}$, and the capacity is provided by

(62) $$C_{\mathrm{iso}}=\lim_{N\to \infty }\frac{1}{N}\log\det\left(\frac{P}{\sigma^{2}}H_{N}H_{N}^{H}+I_{N}\right).$$

It is evident that $C_{\mathrm{iso}}$ is achieved by any input of the form $X_{N}=B_{N}S_{N}$, where $B_{N}$ is a set of orthonormal basis (i.e., $B_{N}^{H}B_{N}=B_{N}B_{N}^{H}=I_{N}$) and $S_{N}$ is a vector of zero-mean i.i.d. Gaussian symbols with covariance $E\left[S_{N}S_{N}^{H}\right]=PI_{N}$. As shown in Section 2, the set of sequence $\left\{v_{n,k}:0\leq n\leq L-1,0\leq k\leq K-1\right\}$ forms an orthonormal basis. Thus, the capacity of the DD channel is provided by (62).

## 7. Conclusions

In this work, we have presented an OTFS based on the discrete Zak transform. The discrete Zak transform-based description allows for an efficient digital implementation of OTFS. Furthermore, we derived the input–output relation for the symbols in the delay-Doppler domain solely based on discrete Zak transform properties, which provides a concise description of OTFS compared to the pre- and postprocessing approaches for OFDM.

Our presented discrete Zak transform approach can be used to study and evaluate OTFS from a different perspectives, potentially leading to OTFS performance improvements. For example, considering Nyquist pulses p(t) with larger roll-off factors allows the interference in the delay domain to be controlled. Additionally, applying windows to the subsampled sequences of the DZT reduces the interference in the Doppler domain.

## Data Availability

Not applicable.

## Appendix A. Proof of Relation (14)

Substituting x[n] in (1) by (13), we obtain

(A1) $$Z_{x}^{\left(L,K\right)}\left[n,k\right]=\frac{1}{K\sqrt{L}}\sum_{l=0}^{K-1}\sum_{k^{\prime }=0}^{KL-1}X\left[k^{\prime }\right]e^{j2\pi \left(\frac{k^{\prime }}{KL}\left(n+lL\right)-\frac{k}{KL}l\right)}.$$

Note that in the derivation of (13), the case for L=1 was considered; thus, the sequence *x* has a period *K*. Here, on the other hand, we consider the sequence *x* to be KL-periodic. Therefore, (13) is adopted accordingly by substituting *K* by KL. Next, we rearrange terms and obtain

(A2) $$Z_{x}^{\left(L,K\right)}\left[n,k\right]=\frac{1}{K\sqrt{L}}\sum_{k^{\prime }=0}^{KL-1}X\left[k^{\prime }\right]e^{j2\pi \frac{k^{\prime }}{KL}n}\sum_{l=0}^{K-1}e^{-j2\pi \frac{k^{\prime }-k}{K}l},$$

where we finally replace the last sum by relation (3) which, due to the sifting property of the Kronecker delta, leads to

(A3) $$Z_{x}^{\left(L,K\right)}\left[n,k\right]=\frac{1}{\sqrt{L}}\sum_{l=0}^{L-1}X\left[k+lK\right]e^{j2\pi \frac{k+lK}{KL}n}.$$

## Appendix B. Proof of Relation (16)

In a first step, we rewrite the summation in (10) as a double summation, i.e.,

(A4) $$X\left[k\right]=\frac{1}{\sqrt{KL}}\sum_{l=0}^{K-1}\sum_{n=0}^{L-1}x\left[n+lL\right]e^{-j\frac{k}{KL}\left(n+lL\right)}.$$

Next, we use relation (19) to express x[n+lL] through its IDZT, which leads to

(A5) $$X\left[k\right]=\frac{1}{K\sqrt{L}}\sum_{l=0}^{K-1}\sum_{n=0}^{L-1}\sum_{k^{\prime }=0}^{K-1}Z\left[n,k^{\prime }\right]e^{-j\frac{k-k^{\prime }}{K}l}e^{-j\frac{k}{KL}n},$$

and in a final step we use relation (3) with respect to the summation over *l*, which results in

(A6) $$X\left[k\right]=\frac{1}{\sqrt{L}}\sum_{n=0}^{L-1}Z_{x}\left[n,k\right]e^{-j2\pi \frac{k}{KL}n}.$$

## Appendix C. Proof of Relation (25)

To prove the relation (25), we substitute the DZT $Z_{x}$ and $Z_{y}^{*}$ by their definition in (1). After rearranging terms, we obtain

(A7) $$\frac{1}{K}\sum_{n=0}^{L-1}\sum_{l^{\prime }=0}^{L-1}\sum_{l^{\prime \prime }=0}^{L-1}x\left[n+l^{\prime }L\right]y^{*}\left[n+l^{\prime \prime }L\right]e^{-j2\pi \frac{l}{L}n}\sum_{k=0}^{K-1}e^{-j2\pi \frac{k}{K}\left(l^{\prime }-l^{\prime \prime }-m\right)}.$$

We can us relation (3) to substitute the last summation. From the sifting property of the Kronecker delta (3), we have

(A8) $$\sum_{n=0}^{L-1}\sum_{l^{\prime }=0}^{L-1}x\left[n+l^{\prime }L\right]y^{*}\left[n+\left(l^{\prime }-m\right)L\right]e^{-j2\pi \frac{l}{L}n}.$$

Because the complex exponential sequence is periodic, with a period *L*, we can rewrite the double summation as a single summation, providing us with

(A9) $$\sum_{n=0}^{KL-1}x\left[n\right]y^{*}\left[n-mL\right]e^{-j2\pi \frac{l}{L}n}$$

which can be recognized as the inner product between *x* and $y_{m,l}$.

## Appendix D. Proof of the Modulation Property

To prove the modulation property, we can use the definition of the sequence z=x·y and the definition of the DZT in (1), which is

(A10) $$Z_{z}\left[n,k\right]=\frac{1}{\sqrt{K}}\sum_{l=0}^{K-1}x\left[n+lL\right]y\left[n+lL\right]e^{-j2\pi \frac{k}{K}l}.$$

Now, expressing x[n+lL] using (19), we have

(A11) $$Z_{z}\left[n,k\right]=\frac{1}{K}\sum_{m=0}^{K-1}Z_{x}\left[n,m\right]\sum_{l=0}^{K-1}y\left[n+lL\right]e^{-j2\pi \frac{\left(k-m\right)}{K}l}.$$

Finally, using the DZT definition (1), we obtain

(A12) $$Z_{z}\left[n,k\right]=\frac{1}{\sqrt{K}}\sum_{m=0}^{K-1}Z_{x}\left[n,m\right]Z_{y}\left[n,k-m\right].$$

## Appendix E. Proof of the Convolution Property

To prove relation (31), we first express the circular convolution as a multiplication in the DFT domain, i.e.,

(A13) $$Z\left[k\right]=\sqrt{KL}X\left[k\right]Y\left[k\right],$$

where the factor $\sqrt{KL}$ is due to the unitary definition of the DFT. Using (14), we have

(A14) $$Z_{z}\left[n,k\right]=\sqrt{K}\sum_{l=0}^{L-1}X\left[k+lK\right]Y\left[k+lK\right]e^{j2\pi \frac{k+lK}{KL}n}.$$

Now, using (16) to express the elements of the DFT through their DZT, we obtain

(A15) $$Z_{z}\left[n,k\right]=\frac{\sqrt{K}}{L}\sum_{n^{\prime }=0}^{L-1}\sum_{n^{\prime \prime }=0}^{L-1}Z_{x}\left[n^{\prime },k\right]Z_{y}\left[n^{\prime \prime },k\right]\sum_{l=0}^{L-1}e^{-j2\pi \frac{k+lK}{KL}\left(n^{\prime }+n^{\prime \prime }-n\right)}.$$

Substituting the last sum by (3) and applying the sifting property of the Kronecker delta, we finally have

(A16) $$Z_{z}\left[n,k\right]=\sqrt{K}\sum_{n^{\prime }=0}^{L-1}Z_{x}\left[n^{\prime },k\right]Z_{y}\left[n-n^{\prime },k\right].$$

## Appendix F. Proof of Theorem 1

To prove Theorem 1, we start by expressing the sequence *y* in (45) as

(A17) $$y=\left(x\cdot u_{\nu_{p}}\right)⊛h_{\tau_{p}},$$

where $u_{\nu_{p}}\left[n\right]=e^{j2\pi (k_{p}/N)n}$. Using the modulation property (30) and the convolution property (31), we can express the DZT of *y* as

(A18) $$Z_{y}\left[n,k\right]=\sum_{m=0}^{L-1}\left(\sum_{l=0}^{K-1}Z_{x}\left[m,l\right]Z_{\nu }\left[m,k-l\right]\right)Z_{\tau }\left[n-m,k\right].$$

Here, $Z_{\nu }$ is the DZT of sequence $u_{\nu }$, which is

(A19) $$\begin{array}{cc} Z_{\nu }\left[n,k\right] & =\frac{1}{\sqrt{K}}\sum_{l=0}^{K-1}e^{j2\pi \frac{k_{p}}{KL}\left(n+lL\right)}e^{-j2\pi \frac{k}{K}l}\\ \; & =\frac{1}{\sqrt{K}}e^{j2\pi \frac{k_{p}}{KL}n}\sum_{l=0}^{K-1}e^{-j2\pi \frac{k-k_{p}}{K}l}\\ \; & =\frac{1}{\sqrt{K}}e^{j2\pi \frac{k_{p}}{KL}n}\frac{1-e^{-j2\pi (k-k_{p})}}{1-e^{-j2\pi \frac{k-k_{p}}{K}}}\\ \; & =\frac{1}{\sqrt{K}}e^{j2\pi \frac{k_{p}}{KL}n}e^{-j\pi \frac{K-1}{K}\left(k-k_{p}\right)}\frac{\sin\left(2\pi (k-k_{p})\right)}{\sin\left(2\pi \frac{k-k_{p}}{K}\right)}. \end{array}$$
